# Natural History of Cutaneous Human Papillomavirus (HPV) Infection in Men: The HIM Study

**DOI:** 10.1371/journal.pone.0104843

**Published:** 2014-09-08

**Authors:** Shalaka S. Hampras, Anna R. Giuliano, Hui-Yi Lin, Kate J. Fisher, Martha E. Abrahamsen, Bradley A. Sirak, Michelle R. Iannacone, Tarik Gheit, Massimo Tommasino, Dana E. Rollison

**Affiliations:** 1 Department of Cancer Epidemiology, Moffitt Cancer Center, Tampa, Florida, United States of America; 2 Department of Biostatistics, Moffitt Cancer Center, Tampa, Florida, United States of America; 3 QIMR Berghofer Medical Research Institute, Cancer and Population Studies, Royal Brisbane Hospital, Brisbane, Queensland, Australia; 4 Infections and Cancer Biology Group, International Agency for Research on Cancer-World Health Organization, Lyon, France; Albert Einstein College of Medicine, United States of America

## Abstract

Accumulating evidence suggests that cutaneous human papillomavirus (HPV) infection is associated with non-melanoma skin cancer (NMSC). Little is known about the natural history of cutaneous HPV. A sub-cohort of 209 men with no NMSC history, initially enrolled in the HPV infection in men (HIM) study, were followed for a median of 12.6 months. Epidemiological data were collected through self-administered questionnaires. Cutaneous HPV DNA was measured in normal skin swabs (SS) and eyebrow hairs (EB) for 25 and 16 HPV types in genera β and γ, respectively. Any β HPV infection was more prevalent in SS (67.3%) compared to EB (56.5%, p = 0.04). Incidence in SS was higher than 20 per 1,000 person-months for HPV types 4, 5, 23, 38 and 76. Median duration of persistence of β and γ HPV infection was 8.6 and 6.1 months in EB, respectively, and 11.3 months and 6.3 months, in SS, respectively. Older age (>44 years vs. 18-30 years) was significantly associated with prevalent (SS OR = 3.0, 95% CI = 1.2–7.0) and persistent β HPV infection (EB OR = 6.1, 95% CI = 2.6–14.1). History of blistering sunburn was associated with prevalent (OR = 2.8, 95% CI = 1.3–5.8) and persistent (OR = 2.3, 95% CI = 1.2–4.6) β HPV infection in SS. Cutaneous HPV is highly prevalent in men, with age and blistering sunburn being significant risk factors for cutaneous β HPV infection.

## Introduction

Human papillomavirus (HPV) is a non-enveloped DNA virus, primarily infecting stratified epithelium [1], [2]. HPV infections are broadly classified as mucosal (α genus) or cutaneous (α, β, γ, μ, Nv genera) [3]. An etiologic role for mucosal HPV infection has been established for several cancers, including penile [4] and cervical [5], [6] as well as a subset of head and neck cancers [7]. In the majority of cervical cancers, viral DNA integrates into the host genome, further facilitating the transformation of infected cells [8], [9]. Accumulating evidence suggests that, infection with cutaneous HPV types is associated with increased risk of non-melanoma skin cancer (NMSC), in both immunocompetent and immunocompromised individuals [10]–[12], perhaps through an indirect mechanism. For example, inhibition of ultraviolet radiation induced apoptosis has been suggested as a potential mechanism of carcinogenic activity of cutaneous HPV [13], [14].

The earliest evidence for oncogenic potential of β HPV types came from studies by Jablonska and colleagues, in 1972, which demonstrated the presence of β HPV types in cutaneous lesions of patients suffering from Epidermodysplasia Verruciformis (EV) [15], a rare hereditary skin disease that often progresses to squamous cell carcinoma (SCC) of skin in solar exposed areas. Cutaneous HPV DNA has been detected in eyebrow hairs, normal skin samples as well as tumor tissues from NMSC cases [16]. Several studies have reported a positive association between cutaneous HPV DNA and/or seropositivity and NMSC [17]–[20]. Despite its potential role in the development of SCC, very little is known about the natural history of cutaneous HPV infection. This information is essential to further elucidate the role of cutaneous HPV in skin cancer and also to guide future preventive measures targeted at reducing the burden of cutaneous HPV infection.

Few studies have evaluated the prevalence and persistence of cutaneous HPV among healthy individuals. Cutaneous HPV is thought to be ubiquitous in skin, with hair follicles serving as the reservoirs of persistent HPV infection [21], [22]. High prevalence of cutaneous HPV in skin of infants and young children, indicates that exposure to HPV occurs very early in life [23], with asymptomatic infection persisting for several years [24]. Prevalence rates ranging from 42% in African population to 70% in European and Asian populations have been reported among healthy adults [25]. Prevalence of over 90% have been reported among immunocompromised patients [22].

In this study, we describe the natural history of cutaneous HPV infection in a cohort of 209 healthy men in Tampa, Florida. We evaluated a spectrum of epidemiological measures including risk factors associated with prevalence, persistence and incidence across two genera of cutaneous HPV infection. We observed that cutaneous HPV is highly prevalent in men and that, age and blistering sunburn are significant risk factors for cutaneous β HPV infection in men.

## Material and Methods

### Study population

The study population for the current analysis comprises a sub-cohort of men who participated in the U.S. site of the HPV infection in men (HIM) study, a large, multi-national prospective cohort study of the natural history of HPV infection in men [26], [27]. The HIM study methods have been described in detail previously [26], [27]. Briefly, between July 2005 and September 2009, study participants were recruited to the HIM study in Tampa, Florida, through mass advertisement targeted to university students, faculty, staff and members of the general population. Inclusion criteria were: 1) men aged 18–70 years; 2) reside in Florida; 3) have had no prior diagnosis of penile or anal cancers; 4) have never been diagnosed with genital and/or anal warts; 5) have not participated in an HPV vaccine study; 6) have no prior diagnosis of HIV/AIDS; 7) have no current penile discharge or burning during urination; 8) were not currently being treated for an STD; 9) have not been imprisoned or homeless during last 6 months; and 10) had not been in a drug or alcohol treatment program over the last 6 months at enrollment. Between November 2008 and June 2010, 1,082 participants in the HIM study were invited to participate in a supplemental study of the natural history of cutaneous HPV infection, requiring additional biospecimen collection. In the parent HIM study, the participants were followed every six months up to four years of follow-up. Nine hundred and sixty seven men enrolled in the parent HIM study had at least one sample of eyebrow hair or skin swab at their baseline visit. Of these 967 men, 965 men (99.8%) had three samples (one sample of eyebrow hair and one skin swab sample each, from sun exposed and unexposed skin), at their baseline visit. 89.4% and 66.7% men had all three samples at two and three visits, respectively. In order to maximize our observation time and facilitate estimation of incidence and persistence, the sub-study was restricted to those men who had all three samples for at least 4 visits (n = 211). Of these, 209 men had viable samples and were included in the final analysis.

### Ethics statement

Written informed consent was obtained from all participants in the parent study, and a separate addendum to the consent was completed by all participants in the sub-study. The parent study and sub-study protocols were approved by institutional review boards at each recruiting site, including the Human Subjects Committees of the University of South Florida, the Ludwig Institute for Cancer Research, São Paulo, Brazil, the Centro de Referencia e Tratamento de Doencas Sexualmente Transmissiveis e AIDS, Brazil, and the National Institute of Public Health of Mexico, as described previously [27].

### Data collection

#### Questionnaires

At the enrollment visit, HIM study participants completed a comprehensive self-administered questionnaire with information on demographics (age, race, education, and marital status), socioeconomic status, medical history, smoking status, alcohol consumption, and sexual history. Additional questions on risk factors for skin cancer (skin's reaction to season's first sun exposure, history of blistering sunburn, etc.) were added to the cutaneous HPV sub-study.

#### Eyebrow hairs and swabs of normal skin

Although, in the parent HIM study, swabs from both sun exposed and unexposed skin were collected, only swabs from sun-exposed skin were used for HPV DNA measurement in the present sub-study due to limited funding. A 5×5 cm area of normal, sun exposed skin, from the top of forearm, was pre-wetted (0.9% NaCl) and swabbed back and forth five times using a cotton-tipped Dacron swab (Digene, Gaithersburg, MD, USA). All swabs were placed in a separate vial and preserved in 500 µl Digene Standard Transport Medium (STM) for HPV DNA testing. Three to four hairs were plucked from each eyebrow (6–8 eyebrow hairs per individual) using disposable tweezers. Hairs with attached follicles were snap frozen in liquid nitrogen. All tissues were stored at −70°C until testing for cutaneous HPV DNA.

#### DNA extraction from skin swabs and eyebrow hair

DNA extraction was performed at the International Agency for Research on Cancer in Lyon, France, using the Qiagen BioRobot EZ1 with the EZ1 DNA tissue kit according to the manufacturer's instructions (Qiagen, Hilden, Germany). Briefly, the Dacron swabs were carefully cut into an Eppendorf tube using scissors and incubated overnight in proteinase K and buffer G2 (Qiagen, Hilden, Germany) at 56°C. An EZ1 DNA Forensic protocol was used to extract the DNA from eyebrow hairs according to the manufacturer's instructions. To monitor the possible occurrence of cross-contamination between the different specimens during DNA extraction, tubes containing buffer only were also included (one tube with buffer every ten specimens).

#### Viral DNA detection using Multiplex PCR/Luminex assay

The detection of viral DNA was performed by a multiplex PCR/Luminex assay [28], followed by the HPV typing using Luminex beads coupled with β or γ HPV type-specific probes. The assay was performed using approximately 100 ng of total DNA and specific primers amplifying a part of the E7 gene for 25 HPV types in genus β (5, 8, 9, 12, 14, 15, 17, 19, 20, 21, 22, 23, 24, 25, 36, 37, 38, 47, 49, 75, 76, 80, 92, 93, 96) and 16 HPV types in genus γ (4,48,50, 60, 65, 88, 95, 101, 103,108,109,112,116,119,121,123) [28], [29]. Although, a large number of HPV types are being continually discovered, a limited coverage of HPV types was offered in the multiplex assay. Two primers for the amplification of β-globin were included in the assay to provide a positive control for the quality of the template DNA. The positivity of the assay was given by the intensity of the fluorescent signal detected by the Luminex apparatus and was expressed as the median fluorescence intensity (MFI) of at least 100 beads per bead set. The cutoff was calculated for each HPV-specific probe by considering the MFI values obtained with no respective PCR product. The cutoff was computed by adding 5 MFI to 1.1× the median background value, as described by Schmitt et al. [30]. All MFI values above the cut-off have been considered positive. PCR was performed with the QIAGEN multiplex PCR kit according to the instructions of the manufacturer. This multiplex PCR protocol is highly sensitive, being able to detect only 10 copies of the viral genome [28], [29]. HPV genotyping was successfully repeated in a blind manner, three times in 10 individual subjects, demonstrating reliability of multiplex PCR for detection of specific HPV types [28]. Similar strategy was used for determining the sensitivity and specificity of γ HPV assay, showing features similar to those of the β HPV assay.

Following PCR amplification, 10 μL of each reaction was analyzed by multiplex genotyping using a Luminex based assay as described [31]. Results were expressed as the median fluorescence intensity (MFI) of at least 100 beads per bead set. Of the 1,385 samples, 1,234 (89%) were β-globin positive. Thirty (2%) β-globin negative and HPV positive samples were included, while 120 (9%) β-globin negative and HPV negative samples were excluded from the analyses. All the tubes containing buffer only were tested negative for HPV DNA and β globin.

### Statistical analysis

Baseline characteristics, including demographics, skin cancer risk factors, lifestyle factors and history of sexually transmitted diseases, were summarized for the cohort using descriptive statistics. HPV prevalence in eyebrow hairs and normal skin swabs was estimated by phylogenetic species along with concurrent infections with other HPV types. Type-specific prevalence of HPV was defined as the proportion of men who were positive for a given HPV out of the baseline cohort with valid samples. Genus- and species-specific prevalence was defined as the proportion of men who were positive for at least one HPV type in the given genus or species. The statistical significance of differences in the prevalence of HPV infection between eyebrow hairs and normal skin swabs was tested using Fisher's exact test. Incidence (in person-months) was determined for any HPV infection, as well as type-specific and species-specific HPV infections in normal skin swabs or eyebrow hairs. For analyses of incidence of any HPV infection, the participant had to be negative for all HPV types (genus β or γ) at baseline, and only the first incidence of any HPV infection was taken into account. Similarly, for species-specific and type-specific HPV infections, the participant had to be negative at baseline for that particular HPV species or type, respectively. Time to incidence of HPV infection was defined as interval, in months, between baseline visit and first visit with a positive HPV sample or time point being censored. Due to fewer events, median time to incidence of γ HPV infection in eyebrow hairs could not be estimated. Time to clearance of HPV infection was defined as interval (in months) between incident HPV infection and clearance of all type-specific HPV infections.

Persistence of any HPV (genus β or γ) or type-specific HPV was defined as any or type-specific HPV infection at ≥2 consecutive visits. For a given type of HPV persistence, only participants who were positive for the HPV genus or type and had at least one follow-up visit after that were included. Kappa coefficient (k) was used to determine the concordance of viral infections across eyebrow hairs and skin swabs for each β-HPV type. The kappa coefficients <0 indicates no agreement, 0–0.20 as slight, 0.21–0.40 as fair, 0.41–0.60 as moderate, 0.61–0.80 as substantial, and 0.81–1 as almost perfect agreement [32].

Logistic regression models were used to estimate age-adjusted odds ratios (OR) and 95% confidence intervals (CI) for the associations between risk factors and prevalence of overall and species-specific HPV infections (β1 and β2) (due to sample size constraints, risk factors associated with type-specific HPV infections were not examined.). Variables that were significantly associated with β HPV prevalence in an age-adjusted model were further evaluated after stratification by β HPV species. Logistic regression was conducted to estimate the association between risk factors and incidence as well as persistence of HPV infection, after adjusting for age. Logistic regression analyses were conducted separately for HPV infection in eyebrow hairs and normal skin swabs. Kaplan-Meier curves were derived for incidence and clearance of any HPV infection. An alpha level of 0.05 was considered statistically significant. Adjustment for multiple comparisons was not conducted. All analyses were conducted using SAS software, version 9.3 (SAS Institute Inc., Cary, NC, USA) and R software, version 2.13.1.

## Results

The median follow up time was 12.6 months. As seen in Table 1, majority (74.5%) of men self-reported their race as White and was between 18 and 30 years of age (51.7%).

**Table 1:** Baseline characteristics of 209 male participants in the humanpapilloma virus infection (HIM) Study, Tampa, Florida.

Variable	n (%)
**Age**	
18–30	108 (51.7)
31–44	44 (21.0)
45+	57 (27.3)
**Self-Identified Race**	
White	155 (74.5)
Other	53 (25.5)
**Spanish/Hispanic/Latino**	
No	177 (84.7)
Yes	32 (15.3)
**Marital status**	
Single, Never Married or Divorced/Separated	148 (70.8)
Married or Cohabiting, Living Together	61 (29.2)
Highest level of education	
**High school or below**	38 (18.2)
Vocational school/Some college	110 (52.6)
Graduated college/Graduate school	61 (29.2)
**Skin reaction to season's first sun exposure**	
No change in skin color	37 (17.9)
Tan with no sunburn	53 (25.6)
Mild sunburn that becomes a tan	80 (38.6)
Sunburn	37 (17.9)
**Ever had a blistering sunburn**	
No	103 (49.7)
Yes	104 (50.2)
**Lifetime number of blistering sunburns**	
None	103 (49.7)
1	38 (18.3)
2	28 (13.5)
3+	38 (18.3)
**Had an alcoholic beverage in the past month**	
No	35 (16.7)
Yes	174 (83.3)
**Number of days drank in past month**	
0	35 (19.9)
1–8	81 (46.0)
9+	60 (34.1)
**Current smoker**	
No	176 (84.2)
Yes	33 (15.8)
**Ever smoker**	
No	123 (59.4)
Yes	84 (40.6)
**Smoking status**	
Never	123 (59.4)
Former	51 (24.6)
Current	33 (15.9)
**Ever been diagnosed with an STD**	
No	180 (86.1)
Yes	29 (13.9)
**Lifetime female vaginal sex partners**	
0–1	42 (20.1)
2–9	61 (30.6)
10+	96 (48.2)
**Female vaginal sex partners in past 6 months**	
None	53 (25.6)
1	105 (50.7)
2+	49 (23.7)
**Lifetime male anal-sex partners**	
None	158 (90.3)
1+	17 (9.7)
**Male anal-sex partners in the past 3 months**	
None	182 (98.9)
1+	2 (1.09)

### HPV DNA in eyebrow hair and normal skin

As seen in Table 2, cutaneous HPV prevalence was higher among normal skin swab samples than eyebrow hairs, particularly for any β HPV type (67.3% in normal skin swabs vs. 56.5% in eyebrow hairs). HPV types in genus β, species 1 (5, 12, 21 and 24) were the most highly prevalent in normal skin swabs and their prevalence in skin swabs was higher compared to that in eyebrow hairs (McNemar's p = 0.004). No significant difference was noted in the prevalence of β2 HPV types between the two sites of infection. Significantly higher prevalence of γ HPV infection was also observed in normal skin swabs (26.8%) compared to eyebrow hairs (15.9%, McNemar's p = 0.002). The concordance between HPV DNA in eyebrow hairs and normal skin swabs varied by HPV type, with kappa values ranging from no agreement (k = −0.022) for HPV type 123 to substantial agreement (k = 0.80) for types 47 in β1, 92 in β4 and 112 in γ HPV (data not shown).

**Table 2:** Prevalence, incidence and persistence of type-specific cutaneous HPV infection at baseline in eyebrow hairs and normal skin swabs of men residing in Tampa, Florida.

	Eyebrow hairs					Normal skin swabs				
HPV type	Prevalence n (%) HPV positive^a^	Incident Cases**	Incidence per 1000 person months	Persistent cases	Persistence§§%	Prevalence n (%) HPV positive^a^	Incident cases**	Incidence per 1000 person months	Persistent cases	Persistence§§ %
Any β*	118 (56.5)	40	37.49	71	48.3	105 (67.3)	50	85.01	95	63.8
β_1_ *	63 (30.1)	43	22.74	41	46.1	72 (46.2)	91	56.95	65	57.5
5*	16 (7.7)	17	6.39	10	43.5	27 (17.3)	42	22.02	21	47.7
8	14 (6.7)	6	2.2	8	42.1	7 (4.5)	21	9	5	62.5
12*	20 (9.6)	11	4.16	14	56.0	31 (19.9)	38	19.53	20	50.0
14	5 (2.4)	4	1.39	1	20.0	6 (3.8)	23	9.96	4	33.3
19	6 (2.9)	4	1.41	1	11.1	4 (2.6)	12	5.11	4	44.4
20	10 (4.8)	9	3.25	6	35.3	5 (3.2)	22	9.54	9	60.0
21*	7 (3.3)	6	2.13	4	33.3	12 (7.7)	27	12.43	13	52.0
24*	17 (8.1)	12	4.58	11	42.3	25 (16)	33	16.72	13	34.2
25	2 (1)	1	0.34	2	66.7	2 (1.3)	7	2.95	5	71.4
36	7 (3.3)	8	2.84	4	36.4	10 (6.4)	15	6.62	4	26.7
47	7 (3.3)	4	1.42	6	66.7	9 (5.8)	15	6.7	8	53.3
93	9 (4.3)	4	1.44	5	41.7	10 (6.4)	22	9.83	7	36.8
β_2_	83 (39.7)	38	24.05	49	44.1	69 (44.2)	95	60.07	57	50.9
9	12 (5.7)	4	1.45	6	40.0	10 (6.4)	32	14.63	6	27.3
15	8 (3.8)	1	0.35	5	55.6	11 (7.1)	15	6.72	7	46.7
17	13 (6.2)	6	2.21	10	52.6	16 (10.3)	34	16.22	11	36.7
22	17 (8.1)	10	3.76	9	37.5	10 (6.4)	29	12.94	9	50.0
23	28 (13.4)	14	5.68	13	35.1	13 (8.3)	48	23.01	16	51.6
37	15 (7.2)	10	3.75	11	45.8	14 (9)	34	15.49	14	66.7
38	32 (15.3)	18	7.57	16	34.8	22 (14.1)	44	22.35	19	44.2
80	7 (3.3)	16	5.77	4	23.5	8 (5.1)	27	12.02	7	38.9
β_3_	36 (17.2)	12	5.06	18	40.0	28 (17.9)	61	24.62	25	65.8
49	5 (2.4)	2	0.7	0	0.0	2 (1.3)	9	3.75	1	33.3
75	6 (2.9)	5	1.76	1	11.1	6 (3.8)	11	4.64	2	40.0
76	29 (13.9)	7	2.83	17	48.6	23 (14.7)	44	21.67	23	63.9
β_4_ (92)	2 (1)	3	1.04	1	25.0	3 (1.9)	5	2.1	3	50.0
β_5_ (96)	7 (3.3)	3	1.07	3	30.0	4 (2.6)	16	6.81	3	42.9
Any □^b^ *	33(15.9)	30	13.02	16	30	56(26.8)	90	49.77	49	51
4*	13(6.2)	16	5.92	5	23	28(13.4)	65	28.49	24	45
48	1(0.5)	2	0.68	1	50	0(0)	14	4.96	2	50
50	13(6.2)	7	2.58	9	45	15(7.2)	32	12.54	15	52
60	0(0)	1	0.34	0	0	0(0)	1	0.35	0	-
65	3(1.4)	10	3.46	1	13	7(3.3)	32	11.94	6	35
88	2(1)	0	0	0	0	2(1)	2	0.71	0	-
95	1(0.5)	0	0	0	0	2(1)	1	0.35	0	-
108	0	0	0	0	-	0	3	1.05	1	100
112	2(1)	0	0	1	50	3(1.4)	7	2.52	4	50
116	0(0)	1	0.34	0	-	3(1.4)	2	0.71	0	-
121	3(1.4)	1	0.34	2	50	7(3.3)	5	1.81	3	38
123	4(1.9)	2	0.69	1	20	5(2.4)	26	9.51	2	18

a Total numbers of men with valid baseline samples that were tested for HPV: n = 209 and n = 208 eyebrow hairs for β and γ HPV testing, respectively, and n = 156 and n = 209 skin swabs from sun-exposed skin for β and γ HPV testing, respectively.

*Statistically significant difference based on McNemar's exact test (p<0.05).

b γ HPV types 101, 103, 108, 109 and 119 were measured in skin and eyebrow hairs but were not detected in our population at baseline.

**Only for men who tested negative for any or type-specific HPV infection at baseline.

§§ out of men who were positive for any or type-specific HPV infection at the first of two or more consecutive visits. γ HPV types 101, 103, 109, 119 were not detected in skin and eyebrow hair. γ HPV types 88 and 95 were not detected in eyebrow hairs but were detected in skin swabs of 2 and 1 incident infections, respectively.

**Figure 1:**
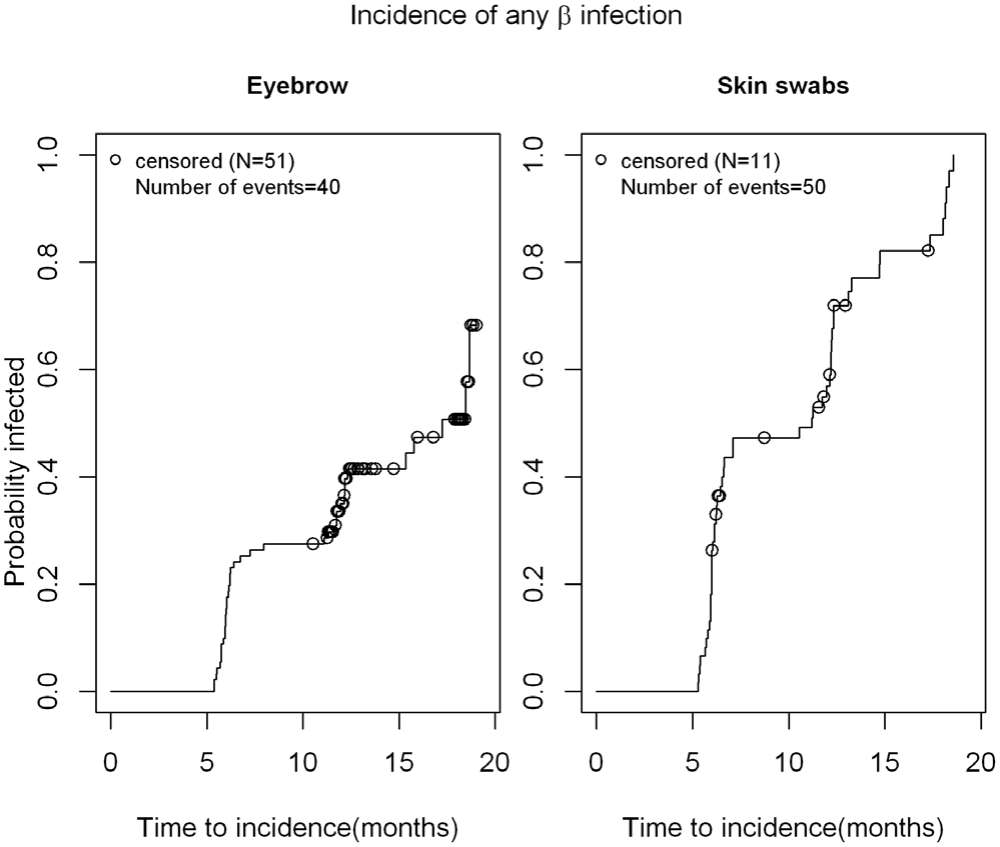
Time to incidence of β-HPV infection in normal skin swabs and eyebrow hairs of men. Kaplan Meier estimate for time to incidence of any β HPV infection in a sample of 209 men. Participants who were negative for all HPV types at baseline were included. Time was counted until their first visit with a HPV positive sample or until censored. The 'last observation carried forward' approach was taken when counting time to incidence (e.g. 0 NA 1 pattern of HPV positivity at consecutive visits, the NA was treated as '0' and the time was counted in the time to incidence.). This only affected skin swab samples since most people were β globin or HPV positive for eyebrows and thus their samples were not removed.

**Figure 2:**
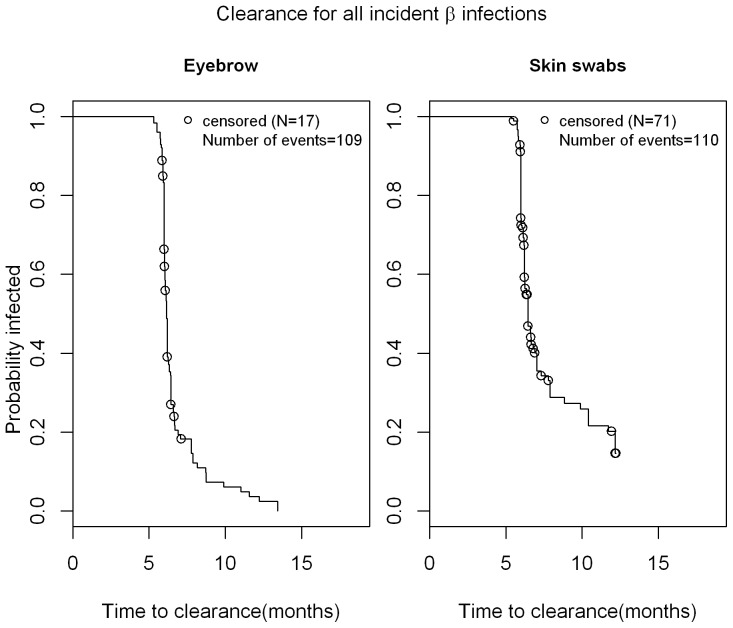
Time to clearance of β-HPV infection in normal skin swabs and eyebrow hairs of men. Kaplan Meier estimate for time to clearance for all type-specific incident infections a study participant (n = 209) had before their last visit with a valid sample. The 'last observation carried forward' approach was taken when counting time to clearance (e.g. for 0 1 NA 0 pattern of HPV positivity, the NA was treated as '1' and the time was counted towards the time to clearance). This only affected skin swab samples since most people were β globin or HPV positive for eyebrows and thus their samples were not removed.

**Figure 3:**
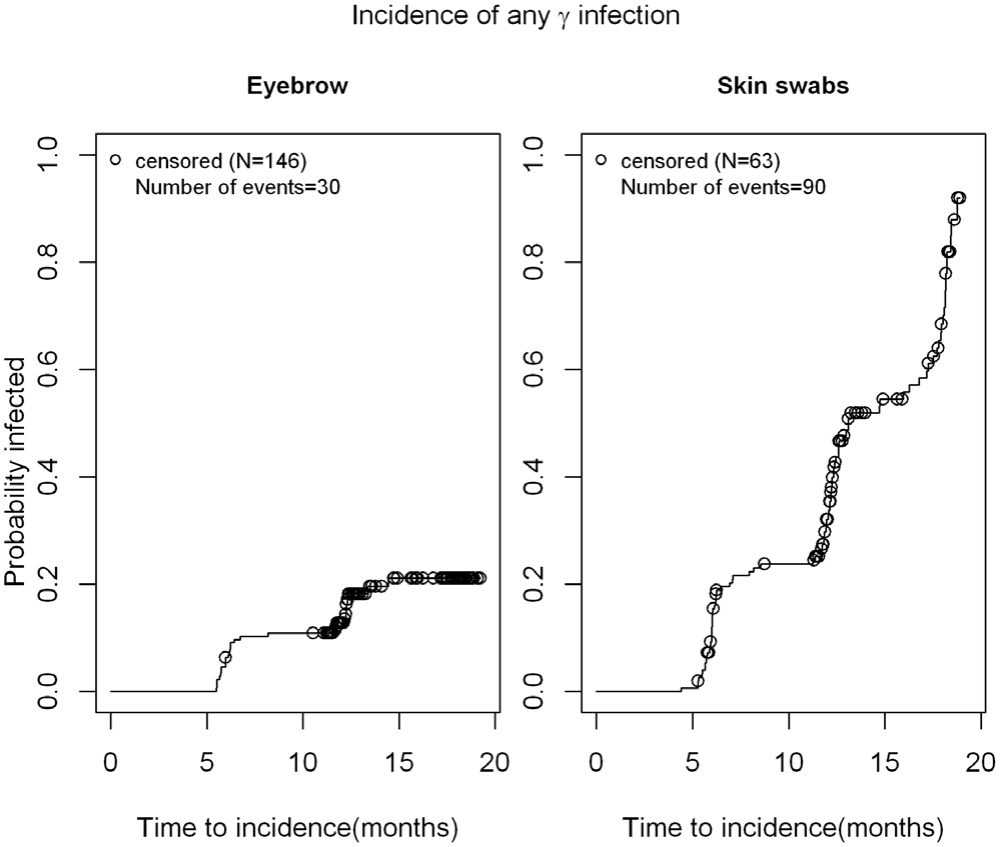
Time to incidence of γ-HPV infection in normal skin swabs and eyebrow hairs of men. Kaplan Meier estimate for time to incidence of any β HPV infection in a sample of 209 men. Participants who were negative for all HPV types at baseline were included. Time was counted until their first visit with a HPV positive sample or until censored. The 'last observation carried forward' approach was taken when counting time to incidence (e.g. 0 NA 1 pattern of HPV positivity at consecutive visits, the NA was treated as '0' and the time was counted in the time to incidence.).

**Figure 4:**
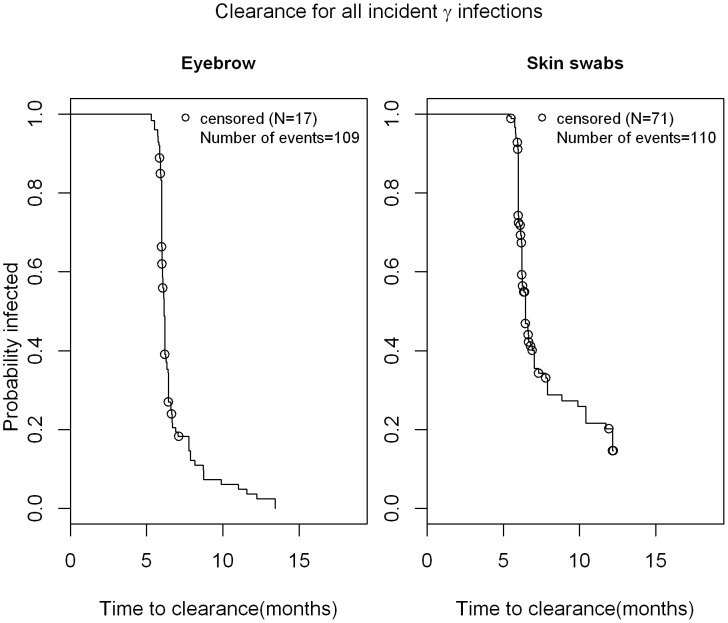
Time to clearance of γ-HPV infection in normal skin swabs and eyebrow hairs of men. Kaplan Meier estimate for time to clearance for all type-specific incident infections a study participant (n = 209) had before their last visit with a valid sample. The 'last observation carried forward' approach was taken when counting time to clearance (e.g. for 0 1 NA 0 pattern of HPV positivity, the NA was treated as '1' and the time was counted towards the time to clearance).

As seen in Figure 1, the median time to incidence of cutaneous β-HPV infection was 17.3 months in eyebrow hairs with a slightly shorter time to incidence in normal skin swabs (11.2 months). Thus, at 12 months, 35% of men had newly acquired cutaneous β-HPV infection in eyebrow hairs while 57% men acquired new cutaneous β-HPV infection in skin swabs. The median time to clearance for any β HPV infection was 6.1 months in eyebrow hairs and 6.44 months in normal skin swabs (Figure 2). For γ HPV infection, the median time to incidence in normal skin swabs was 13.1 months (Figure 3), while the median time to clearance in both, eyebrow hairs and normal skin swabs, was 6.1 months and 6.4 months, respectively (Figure 4).

Incidence and persistence of cutaneous HPV are presented in Table 2. In general, incidence and persistence rates were higher in normal skin swabs compared to those in eyebrow hairs for β and γ HPV infection. The top five HPV types with highest incidence of infection in eyebrow hairs were 38 (β2), 5 (β1), 4 (γ), 80 (β2) and 23 (β2), ranging from 5.7 to 7.6 cases per 1000 person months; while in normal skin swabs these included 4 (γ), 23 (β2), 38 (β2), 5 (β1) and 76(β3), ranging from 21.7 to 28.5 cases per 1000 person months. More than 50% of men had persistence of HPV types 25, 47, 12, 15 and 17 in eyebrow hairs and persistence of HPV types 20, 21, 47, 23, 50, 108, 25, 37, 76 and 8 in skin swabs. Median duration of persistence of β and γ HPV infection was 8.6 and 6.1 months in eyebrow hairs, respectively and 11.3 months and 6.3 months, in skin swabs, respectively.

### Risk factors associated with prevalence, incidence and persistence of HPV infection

Men aged >44 years were more than twice and thrice as likely to have prevalent baseline β HPV infection in eyebrow hairs and skin swabs, respectively, compared to men aged 18–30 years (Table 3). A positive history of blistering sunburn was associated with a more than two-fold increased prevalence of β HPV infection in normal skin swabs, although the association between frequency of sunburns and HPV prevalence did not reach statistical significance (Table 3). No other demographic, lifestyle or sexual history related factors were associated with prevalence of HPV.

**Table 3:** Associations between baseline characteristics and genus β HPV prevalence in eyebrow hairs and normal skin swabs of men residing in Tampa, Florida.

	Eyebrow hairs			Normal skin swabs		
Variable	Total (n = 209)	Any β infection (n = 118) n (%)	Age adjusted OR (95 %CI)	Total (n = 156)	Any β infection (n = 105) n (%)	Age adjusted OR (95 %CI)
Age*						
18–30	108	52 (48.1)	1.0 (reference)	72	44 (61.1)	1.0 (reference)
31–44	44	27 (61.4)	1.7 (0.84–3.5)	33	19 (57.6)	0.9 (0.37–2)
>44	57	39 (68.4)	**2.3 (1.2**–**4.6)**	51	42 (82.4)	**3.0 (1.2**–**7.0)**
**Race**						
White	155	87 (56.1)	1.0 (reference)	115	78 (67.8)	1.0 (reference)
Other	53	30 (56.6)	1.1 (0.6–2.0)	40	26 (65.0)	0.9 (0.4–2.0)
**Spanish/Hispanic/Latino**						
No	177	99 (55.9)	1.0 (reference)	132	89 (67.4)	1.0 (reference)
Yes	32	19 (59.4)	1.3 (0.6–2.9)	24	16 (66.7)	1.2 (0.4–3.0)
**Marital status**						
Single, Never Married or Divorced/Separated	148	82 (55.4)	1.0 (reference)	106	66 (62.3)	1.0 (reference)
Married or Cohabiting, Living Together	61	36 (59)	0.9 (0.5–1.7)	50	39 (78)	1.9 (0.9–4.4)
**Highest level of education**						
High school or below	38	26 (68.4)	1.0 (reference)	28	17 (60.7)	1.0 (reference)
Vocational school/Some college	110	59 (53.6)	0.6 (0.3–1.4)	81	57 (70.4)	1.8 (0.7–4.5)
Graduated college/Graduate school	61	33 (54.1)	0.4 (0.9–1.0)	47	31 (66.0)	1.0 (0.4–2.8)
**Skin reaction to season's first sun exposure**						
No change in skin color	37	20 (54.1)	1.0 (reference)	30	24 (80)	1.0 (reference)
Tan with no sunburn	53	26 (49.1)	1.0 (0.4–2.2)	41	28 (68.3)	0.6 (0.2–1.9)
Mild sunburn that becomes a tan	80	46 (57.5)	1.2 (0.6–2.7)	62	36 (58.1)	0.4 (0.1–1.0)
Sunburn	37	25 (67.6)	1.7 (0.7–4.6)	21	17 (81)	0.9 (0.2–3.9)
**Ever had a blistering sunburn**						
No	103	54 (52.4)	1.0 (reference)	78	45 (57.7)	1.0 (reference)
Yes	104	63 (60.6)	1.3 (0.8–2.4)	76	60 (78.9)	**2.8 (1.3**–**5.8)**
**Lifetime number of blistering sunburns**						
None	103	54 (52.4)	1.0 (reference)	78	45 (57.7)	1.0 (reference)
1	38	24 (63.2)	1.7 (0.8–3.7)	27	21 (77.8)	3.0 (1.0–8.4)
2	28	16 (57.1)	1.2(0.5–2.9)	23	17 (73.9)	2.3 (0.8–6.8)
>2	38	23 (60.5)	1.1 (0.5–2.5)	26	22 (84.6)	3.2 (0.9–10.9)
**Had an alcoholic beverage in the past month**						
No	35	19 (54.3)	1.0 (reference)	27	18 (66.7)	1.0 (reference)
Yes	174	99 (56.9)	1.1(0.5–2.4)	129	87 (67.4)	1.2 (0.5–2.9)
**Number of days drank alcohol in past month**						
0	35	19 (54.3)	1.0 (reference)	27	18 (66.7)	1.0 (reference)
1–8	81	48 (59.3)	1.3 (0.6–3.0)	57	39 (68.4)	1.3 (0.4–3.6)
9+	60	31 (51.7)	0.9 (0.4–2.1)	43	30 (69.8)	1.4 (0.5–4.2)
**Current smoker**						
No	176	96 (54.5)	1.0 (reference)	131	89 (67.9)	1.0 (reference)
Yes	33	22 (66.7)	1.4 (0.6–3.1)	25	16 (64)	0.7(0.3–1.9)
**Ever smoker**						
No	123	64 (52)	1.0 (reference)	88	59 (67)	1.0 (reference)
Yes	84	53 (63.1)	1.2 (0.7–2.3)	67	45 (67.2)	0.7 (0.3–1.6)
**Smoking status**						
Never	123	64 (52)	1.0 (reference)	88	59 (67)	1.0 (reference)
Former	51	31 (60.8)	1.1 (0.6–2.3)	42	29 (69)	0.8 (0.3–1.9)
Current	33	22 (66.7)	1.5 (0.6–3.4)	25	16 (64)	0.7 (0.2–1.9)
**Ever been diagnosed with an STD**						
No	180	98 (54.4)	1.0 (reference)	131	87 (66.4)	1.0 (reference)
Yes	29	20 (69)	1.4 (0.6–3.4)	25	18 (72)	0.9 (0.3–2.6)
**Lifetime female vaginal sex partners**						
0–1	42	23 (54.8)	1.0 (reference)	27	16 (59.3)	1.0 (reference)
2–9	61	37 (60.7)	1.1 (0.5–2.5)	40	29 (72.5)	1.6 (0.5–4.5)
10+	96	56 (58.3)	0.6 (0.2–1.4)	82	57 (69.5)	0.9 (0.3–2.7)
**Female vaginal sex partners in past 6 months**						
None	53	28 (52.8)	1.0 (reference)	38	27 (71.1)	1.0 (reference)
1	105	65 (61.9)	1.6 (0.8–3.2)	81	63 (77.8)	1.7 (0.7–4.1)
2+	49	25 (51)	1.2 (0.5–2.8)	35	15 (42.9)	0.4 (0.1–1.2)
**Lifetime male anal-sex partners**						
None	158	88 (55.7)	1.0 (reference)	118	81 (68.6)	1.0 (reference)
1+	17	12 (70.6)	1.7 (0.6–5.2)	14	10 (71.4)	1.1 (0.3–4.0)

OR = odds ratio, CI = confidence interval. *Unadjusted logistic regression. All analyses, except for age, were adjusted for age using logistic regression.

Evaluation of species-specific β HPV prevalence indicated that men aged >44 years were twice more likely to harbor β1 and β2 types of HPV in eyebrow hairs and more than threefold likely to have β1 and β2 types of HPV infection in normal skin swabs (Table 4), compared to men ages 18–30 years. Similarly, a positive history of blistering skin burns was associated with more than two fold (OR = 2.6, 95% CI = 1.2–5.7) and more than threefold (OR = 3.1, 95% CI = 1.4–7.0) increased prevalence of β1 and β2 HPV in normal skin swabs, respectively. This association was stronger for β2 HPV infection in normal skin swabs (OR = 5.0, 95% CI = 1.4–17.7) among individuals with a history of more than 2 lifetime episodes of sunburns compared to those who reported none. Overall, while the association of these factors with β HPV infection did not vary significantly by HPV species, age and history of blistering sunburns appeared to have strong positive associations with β HPV infection, particularly in normal skin swabs.

**Table 4:** Association between baseline characteristics and prevalence of species-specific HPV infection in men residing in Tampa, Florida.

	Eyebrow hairs		Skin swabs	
	β1 positive n = 63 Age Adjusted OR(95% CI)*	β2 positive n = 83 Age Adjusted OR(95% CI)*	β1 positive n = 72 Age Adjusted OR(95% CI)	β2 positive n = 69 Age Adjusted OR(95% CI)
**Age**				
18–30	1.0(reference)	1.0(reference)	1.0(reference)	1.0(reference)
31–44	1.8 (0.8–4.1)	1.7 (0.8–3.8)	1.0 (0.4–2.4)	1 (0.4–2.5)
>44	**2.7 (1.3**–**6.0)**	**2.4 (1.2**–**5.0)**	**3.1 (1.2**–**7.7)**	**3.6 (1.4**–**9.0)**
**Skin reaction to season's first sun exposure**				
No change in skin color	-	-	1.0(reference)	1.0(reference)
Tan with no sunburn	-	-	0.7 (0.2–2.4)	0.4 (0.1–1.4)
Mild sunburn that becomes a tan	-	-	0.4(0.1–1.1)	0.4 (0.1–1.3)
Sunburn			1.0 (0.2–4.5)	0.8 (0.2–3.9)
**Ever had a blistering sunburn**				
No	1.0(reference)	1.0(reference)	1.0(reference)	1.0(reference)
Yes	1.3 (0.69–2.6)	1.3 (0.7–2.4)	**2.6 (1.2**–**5.7)**	**3.1 (1.4**–**7.0)**
**Lifetime number of blistering sunburns**				
None	1.0(reference)	1.0(reference)	1.0(reference)	1.0(reference)
1	1.6 (0.6–4.1)	1.4 (0.6–3.4)	2.5 (0.8–7.7)	2.7 (0.8–8.5)
2	1.9 (0.8–5.0)	1.0 (0.4–2.6)	2.6 (0.9–8.0)	2.3 (0.7–7.4)
>2	0.7 (0.2–2.0)	1.4 (0.6–3.1)	2.7 (0.7–10.0)	5.0 (1.4–17.7)
**Female vaginal sex partners in past 6 months**				
None	-	-	1.0(reference)	1.0(reference)
1	-	-	1.9 (0.7–5.1)	1.6(0.6–4.3)
2+	-	-	0.5 (0.1–1.4)	0.4 (0.1–1.2)

OR = odds ratio, CI = confidence interval. These factors were selected based on significance in the multinomial logistic regression models.

No significant associations were seen between baseline factors and incident β HPV infection (Table 5) and prevalence or incidence of γ HPV infection, in eyebrow hairs or normal skin swabs (Table 6).

**Table 5:** Associations of baseline characteristics with incidence and persistence for any type of β HPV infection in men residing in Tampa, Florida.

	Incidence		Persistence	
	Eyebrow hairs Age Adjusted OR(95% CI) n = 40	Normal skin swabs Age Adjusted OR(95% CI) n = 50	Eyebrow hairs Age Adjusted OR(95% CI) n = 71	Normal skin swabs Age Adjusted OR(95% CI) n = 95
**Age** *				
18–30	1.0 (referent)	1.0 (referent)	1.0 (referent)	1.0 (referent)
31–44	0.9 (0.3–2.8)	0.5 (0.1–2.04)	2.0 (0.8–4.6)	2.4 (0.9–6.7)
>44	0.4 (0.1–1.3)	0.8 (0.1–4.6)	**6.1 (2.6**–**14.1)**	1.2 (0.6–2.4)
**Self-Identified Race**				
White	1.0 (referent)	1.0 (referent)	1.0 (referent)	1.0 (referent)
Other	1.3 (0.5–3.3)	0.3 (0.1–1.1)	0.58 (0.25–1.34)	0.44 (0.2–0.99)
**Spanish/Hispanic/Latino**				
No	1.0 (referent)	1.0 (referent)	1.0 (referent)	1.0 (referent)
Yes	0.6 (0.1–2.02)	2.6 (0.3–23.5)	1.04 (0.4–2.7)	0.8 (0.3–1.9)
**Marital status**				
Single, Never Married or Divorced/Separated	1.0 (referent)	1.0 (referent)	1.0 (referent)	1.0 (referent)
Married or Cohabiting, Living Together	1.6(0.6–4.4)	0.6 (0.1–2.7)	1.4 (0.6–3.0)	0.9 (0.4–1.8)
**Highest level of education**				
High school or below	1.0 (referent)	1.0 (referent)	1.0 (referent)	1.0 (referent)
Vocational school/Some college	4.01 (0.8–20.5)	3.05 (0.6–14.9)	1.5 (0.6–3.9)	1.7 (0.7–4.1)
Graduated college/Graduate school	5.9 (1.03–34.2)	1.6 (0.3–7.9)	1.7 (0.6–5.0)	1.2 (0.4–3.4)
**Skin reaction to season's first sun exposure**				
No change in skin color	1.0 (referent)	1.0 (referent)	1.0 (referent)	1.0 (referent)
Tan with no sunburn	0.8 (0.2–2.8)	3.1 (0.5–19.2)	0.8 (0.2–2.4)	1.1 (0.4–3.1)
Mild sunburn that becomes a tan	0.8 (0.2–2.5)	3 (0.6–14.9)	0.9 (0.3–2.6)	1.1 (0.4–2.9)
Sunburn	0.7 (0.2–3.5)	1.4 (0.2–12.02)	0.8 (0.2–2.5)	1.4 (0.4–4.5)
**Ever had a blistering sunburn**				
No	1.0 (referent)	1.0 (referent)	1.0 (referent)	1.0 (referent)
Yes	1.1 (0.5–2.7)	1.58 (0.43–5.89)	1.45 (0.72–2.93)	2.27 (1.13–4.54)
**Lifetime number of blistering sunburns**				
None	1.0 (referent)	1.0 (referent)	1.0 (referent)	1.0 (referent)
1	0.4 (0.1–1.7)	2.4 (0.2–23.3)	0.6 (0.2–1.7)	0.9 (0.4–2.2)
2	1.2 (0.3–4.3)	0.5 (0.1–2.5)	4.01 (1.3–12.2)	5.04 (1.3–18.9)
>2	2.7 (0.8–9.9)	Could not be estimated	1.5 (0.6–4.0)	5.5 (1.7–18.5)
**Had an alcoholic beverage in the past month**				
No	1.0 (referent)	1.0 (referent)	1.0 (referent)	1.0 (referent)
Yes	1.0 (0.3–2.9)	0.4 (0.04–3.1)	0.7(0.2–1.8)	0.6 (0.2–1.5)
**Number of days drank in past month**				
0	1.0 (referent)	1.0 (referent)	1.0 (referent)	1.0 (referent)
'1–8	1.1 (0.3–3.6)	0.4 (0.04–3.5)	0.5 (0.2–1.5)	0.4 (0.1–1.1)
9+	0.7 (0.2–2.6)	0.3 (0.03–3.9)	0.9 (0.3–2.9)	1.4 (0.5–4.2)
**Current smoker**				
No	1.0 (referent)	1.0 (referent)	1.0 (referent)	1.0 (referent)
Yes	0.5 (0.1–2.05)	0.8 (0.2–3.7)	0.9 (0.3–2.4)	0.4 (0.1–1.02)
**Ever smoker**				
No	1.0 (referent)	1.0 (referent)	1.0 (referent)	1.0 (referent)
Yes	0.7 (0.3–1.7)	0.7 (0.2–2.4)	1.06 (0.5–2.3)	1.0 (0.5–2.1)
**Smoking status**				
Never	1.0 (referent)	1.0 (referent)	1.0 (referent)	1.0 (referent)
Former	0.8 (0.3–2.2)	0.7 (0.2–2.9)	1.1 (0.5–2.8)	1.7 (0.7–4.3)
Current	0.5 (0.1–2.02)	0.7 (0.1–3.5)	1.0 (0.3–2.7)	0.5 (0.2–1.3)
**Ever been diagnosed with an STD**				
No	1.0 (referent)	1.0 (referent)	1.0 (referent)	1.0 (referent)
Yes	2.1 (0.5–9.3)	1.3 (0.2–7.5)	0.9 (0.3–2.4)	0.5 (0.2–1.5)
**Lifetime female vaginal sex partners**				
0–1	1.0 (referent)	1.0 (referent)	1.0 (referent)	1.0 (referent)
2–9	0.7 (0.2–2.5)	1.9 (0.3–12.7)	1.4 (0.5–3.7)	0.8 (0.3–2.3)
10+	0.7 (0.2–2.2)	1.1 (0.2–5.2)	0.9 (0.3–2.6)	0.8 (0.2–2.4)
**Female vaginal sex partners in past 6 months**				
None	1.0 (referent)	1.0 (referent)	1.0 (referent)	1.0 (referent)
1	2.1 (0.7–6.2)	0.1 (0.01–1.3)	1.0 (0.4–2.4)	1.3 (0.6–3.03)
2+	1.5 (0.4–5.0)	0.3 (0.03–3.5)	0.9 (0.3–2.7)	1.5 (0.5–4.4)

OR = odds ratio, CI = confidence interval.

*Unadjusted logistic regression. All analyses, except for ‘age’ are adjusted for age.

**Table 6:** Association of baseline characteristics with prevalence, incidence and persistence of γ HPV infection in men residing in Tampa, Florida.

	Prevalence		Incidence		Persistence	
	Eyebrow hairs n = 33	Normal skin swabs n = 56	Eyebrow hairs n = 30	Normal skin swabs n = 90	Eyebrow hairs n = 16	Normal skin swabs n = 49
	Age Adjusted OR(95% CI)	Age Adjusted OR(95% CI)	Unadjusted OR(95%CI)	Unadjusted OR(95%CI)	Unadjusted OR(95%CI)	Unadjusted OR(95%CI)
**Age**						
18–30	1.0 (referent)	1.0 (referent)	1.0 (referent)	1.0 (referent)	1.0 (referent)	1.0 (referent)
31–44	0.6 (0.2–1.6)	0.7 (0.3–1.6)	0.3 (0.1–1.0)	0.3 (0.1–0.6)	2.1 (0.4–11.3)	0.5 (0.1–1.5)
>44	0.7 (0.29–1.7)	1.1 (0.5–2.2)	0.6 (0.2–1.4)	0.4 (0.2–0.9)	2.7 (0.7–10.3)	0.7 (0.3–1.8)
**Self-Identified Race**						
White	1.0 (referent)	1.0 (referent)	1.0 (referent)	1.0 (referent)	**	1.0 (referent)
Other	0.6 (0.2–1.6)	0.6 (0.3–1.2)	0.8 (0.3–2.01)	0.7 (0.4–1.5)		0.4 (0.2–1.2)
**Spanish/Hispanic/Latino**						
No	1.0 (referent)	1.0(referent)	1.0(referent)	1.0(referent)	1.0(referent)	1.0(referent)
Yes	0.7 (0.2–2.1)	0.9 (0.4–2.1)	1.4 (0.5–3.8)	0.8 (0.3–1.9)	1.2(0.2–7.4)	0.9 (0.3–3.2)
**Marital status**						
Single, Never Married or Divorced/Separated	1.0(referent)	1.0(referent)	1.0(referent)	1.0(referent)	1.0(referent)	1.0(referent)
Married or Cohabiting, Living Together	0.5 (0.2–1.4)	0.6 (0.3–1.4)	0.6 (0.2–1.5)	1.0 (0.5–1.9)	1.5 (0.4–6.0)	0.5 (0.2–1.2)
**Highest level of education**						
High school or below	1.0(referent)	1.0(referent)	1.0(referent)	1.0(referent)	1.0(referent)	1.0(referent)
Vocational school/Some college	0.7 (0.3–1.8)	1.5 (0.633.8)	1.8 (0.6–5.9)	1.5 (0.6–3.5)	1.4 (0.3–6.1)	1.4 (0.5–4.1)
Graduated college/Graduate school	0.4 (0.1–1.4)	1.2 (0.4–3.1)	0.8 (0.2–3.1)	0.5 (0.2–1.2)	1.3 (0.2–8.4)	0.8 (0.2–3.05)
**Skin reaction to season's first sun exposure**						
No change in skin color	1.0(referent)	1.0(referent)	1.0(referent)	1.0(referent)	1.0(referent)	1.0(referent)
Tan with no sunburn	0.9 (0.3–3.2)	0.6 (0.2–1.7)	1.7 (0.5–5.5)	0.9 (0.3–2.6)	1.0 (0.1–7.4)	2.5 (0.7–9.0)
Mild sunburn that becomes a tan	1.3 (0.4–3.9)	1.45 (0.6–3.4)	1.3 (0.4–4.1)	0.8 (0.3–2.12)	0.8 (0.1–5.2)	2.2 (0.7–7.05)
Sunburn	1.5 (0.4–5.3)	0.73 (0.2–2.1)	0.2 (0.02–1.7)	0.3 (0.1–0.9)	2.5 (0.3–21.4)	3.2 (0.7–14.1)
**Ever had a blistering sunburn**						
No	1.0(referent)	1.0(referent)	1.0(referent)	1.0(referent)	1.0(referent)	1.0(referent)
Yes	0.9 (0.4–1.9)	1.1 (0.6–2.0)	0.6 (0.3–1.3)	1.0 (0.5–1.9)	3.2 (0.9–10.8)	0.8 (0.4–1.9)
**Lifetime number of blistering sunburns**						
None	1.0(referent)	1.0(referent)	1.0(referent)	1.0(referent)	1.0(referent)	1.0(referent)
1	0.7 (0.2–2.1)	1.5 (0.7–3.3)	0.2 (0.05–1.1)	1.2 (0.5–3.1)	1.7 (0.3–10.8)	0.5 (0.2–1.4)
2	0.6 (0.2–2.2)	0.9 (0.4–2.5)	0.5 (0.1–1.9)	1.7 (0.6–4.9)	6.2 (0.8–46.1)	1.0 (0.3–3.5)
>2	1.5 (0.6–4.05)	0.8 (0.3–2.02)	1.1 (0.4–3.1)	0.6 (0.2–1.3)	3.5 (0.8–15.3)	1.5 (0.5–4.7)
**Had an alcoholic beverage in the past month**						
No	1.0(referent)	1.0(referent)	1.0(referent)	1.0(referent)	1.0(referent)	1.0(referent)
Yes	0.6 (0.2–1.4)	1.3 (0.6–3.2)	0.9 (0.3–2.6)	0.5 (0.2–1.3)	0.7 (0.2–2.7)	1.4 (0.5–4.2)
**Number of days drank in past month**						
0	1.0(referent)	1.0(referent)	1.0(referent)	1.0(referent)	1.0(referent)	1.0(referent)
1–8	0.5 (0.2–1.5)	1.1 (0.4–2.8)	0.6 (0.2–2.1)	0.5 (0.2–1.3)	0.5 (0.1–2.8)	1.0 (0.3–3.4)
9+	0.9 (0.3–2.4)	1.4 (0.5–3.7)	1.5 (0.5–4.7)	0.8 (0.3–2.2)	0.8 (0.2–3.7)	2.4 (0.7–8.5)
**Current smoker**						
No	1.0(referent)	1.0(referent)	1.0(referent)	1.0(referent)	1.0(referent)	1.0(referent)
Yes	1.1 (0.4–3.2)	0.7 (0.3–1.85)	1.1 (0.4–3.1)	0.9 (0.4–2.2)	1.2 (0.3–5.7)	1.5 (0.5–4.7)
**Ever smoker**						
No	1.0(referent)	1.0(referent)	1.0(referent)	1.0(referent)	1.0(referent)	1.0(referent)
Yes	1.4 (0.6–3.2)	0.8 (0.4–1.6)	1.2 (0.5–2.6)	0.8 (0.4–1.6)	3.1 (0.9–10.4)	1.1 (0.5–2.5)
**Smoking status**						
Never	1.0(referent)	1.0(referent)	1.00(referent)	1.00(referent)	1.0(referent)	1.0(referent)
Former	1.48 (0.6–3.7)	0.86 (0.4–1.9)	1.2 (0.5–3.1)	0.8 (0.4–1.7)	**4 (1.01**–**15.9)**	0.9 (0.3–2.4)
Current	1.31 (0.4–4.1)	0.7 (0.3–1.9)	1.1 (0.38–3.37)	0.9 (0.4–2.1)	2.0 (0.4–10.4)	1.5 (0.5–4.8)
**Ever been diagnosed with an STD**						
No	1.0(referent)	1.0(referent)	1.0(referent)	1.0(referent)	**	1.0(referent)
Yes	0.6 (0.2–2.4)	1.05 (0.41–2.6)	0.2 (0.02–1.3)	0.7 (0.3–1.9)		0.2 (0.04–0.9)
**Lifetime female vaginal sex partners**						
0–1	1.0(referent)	1.0(referent)	1.0(referent)	1.0(referent)	1.0(referent)	1.0(referent)
2–9	1.4 (0.5–3.9)	0.4 (0.2–1.1)	0.3 (0.1–1.1)	0.7 (0.3–1.9)	1.82 (0.4–9.3)	0.5 (0.1–1.6)
10+	0.6 (0.2–2.1)	1.0 (0.4–2.4)	0.6 (0.2–1.6)	0.6 (0.2–1.5)	1.7 (0.3–8.1)	0.4 (0.1–1.1)
**Female vaginal sex partners in past 6 months**						
None	1.0(referent)	1.0(referent)	1.0(referent)	1.0(referent)	1.0(referent)	1.0 (referent)
1	0.4 (0.2–1.0)	1.4 (0.6–3.0)	0.8 (0.3–2.1)	1.1 (0.5–2.3)	0.3 (0.1–1.1)	0.7(0.2–1.9)
2+	0.4 (0.2–1.3)	0.9 (0.4–2.4)	1.5 (0.5–4.3)	1.3 (0.5–3.1)	0.3 (0.05–1.4)	0.9 (0.3–2.8)

OR = odds ratio, CI = confidence interval.

**No subjects left in the ‘Other’ racial group and in the ‘diagnosed with STD' group. The sample size was too small to conduct age-adjusted analyses of incidence and persistence of γ HPV infection.

Findings on factors associated with persistence of β HPV infection were similar to those of prevalent infections described above. Age>44 years was associated with a six fold (OR = 6.1, 95%CI = 2.6–14.2) increased persistence of infection with β HPV in eyebrow hairs (Table 5) but not in normal skin swabs. History of blistering skin burn was associated with persistence of β HPV in normal skin swabs (OR = 2.3, 95% CI = 1.2–4.6), with increasing frequency of sunburns showing stronger association (Table 5). When restricted to White men, history of blistering sunburns was associated with 2.7 times increased persistence of any HPV infection in normal skin swabs (OR = 2.73, 95% CI = 1.19–6.26), after adjusting for age (data not shown). As expected, this association was stronger than that observed for the overall population (OR = 2.3, 95% CI = 1.2–4.6) (Table 5). However, when analyses were restricted to White men and stratified by HPV species, there were no significant associations between history of blistering sunburns and persistence of β1 (OR = 0.87, 95% CI = 0.29–2.62) or β2 (OR = 1.33, 95% CI = 0.54–3.27) infection in normal skin swabs, after adjusting for age (data not shown).

No significant associations were observed between baseline characteristics and persistence of γ HPV except a significantly increased persistence (OR = 4.0, 95% CI = 1.0–15.9) in eyebrow hairs among former smokers compared to never smokers (Table 6).

## Discussion

In this HIM study sub-cohort of 209 healthy men, we observed a high prevalence of cutaneous β HPV and γ HPV in normal skin swabs, higher than that in eyebrow hair samples. Although cutaneous HPV seroprevalence rates have been reported previously [33]–[35], seroreactivity is not a direct estimate of HPV infection. Hence, we attempted to directly measure type and species-specific HPV DNA in normal skin swabs and eyebrow hairs.

Results presented here contrast to that of other studies which reported a higher prevalence of cutaneous HPV in eyebrow hairs among individuals without a history of skin cancer [36], [37] and lower prevalence (13–37%) in skin [37], [38]. Tarmorshuizen et al. reported a prevalence of 54% in eyebrow hairs among healthy controls [39], which was similar to our findings. Differences across studies could be a reflection of variation in number of HPV types examined. For example, while we evaluated 41 HPV types (25 HPV types from genus β and 16 HPV types from genus γ), previous studies have evaluated fewer HPV types within each genus (6 to 28 HPV types overall across studies) [36]–[39]. Apart from this, methodological differences or even differences in geographical location of study populations may explain the inconsistencies in HPV prevalence across studies. Indeed, HPV prevalence rate has been shown to vary by country [25], [36]. In our study, while we observed a strong association between history of blistering sunburn and both prevalence and persistence of β HPV, no association was seen between tanning ability and β HPV infection, indicating that exposure to ultraviolet (UV) radiation rather than host genetics may be more important determinants of cutaneous HPV infection. Thus, geographical variation in UV radiation might have contributed to variable prevalence estimates in the literature. While we were unable to measure HPV prevalence in sun-unexposed skin, our previous study of NMSC cases indicates that the prevalence of β-HPV is higher in sun-exposed skin swabs (100% prevalence) compared to sun-unexposed swabs (95% prevalence) among NMSC cases [16].

Older age and history of blistering sunburns were significantly associated with both prevalence and persistence of HPV but not with incidence of cutaneous HPV infection. Our findings are consistent with previous reports on the association between age and prevalence of HPV [36], [39]. A positive history of blistering sunburn was associated with a more than two fold increased prevalence of β HPV infection in skin, with a stronger association for β2 HPV infection. While an inverse association between frequency of sunburns and β HPV seropositivity has been reported previously [33], we found a positive association between lifetime history of one blistering sunburn and prevalent β HPV infection in normal skin swabs. We also observed a strong association between frequency of blistering sunburns and persistence of β HPV infection, likely explaining the elevated prevalence observed here. UV exposure mediated immunosuppression may predispose individuals to a higher risk of cutaneous HPV persistence and hence prevalence [40]. Indeed, higher prevalence of cutaneous HPV has been observed among individuals who reported working outdoors for longer duration [41].

The findings of our study should be interpreted with caution. The external validity of these findings is limited to men. Future studies should include women, especially given the previously reported differences in seroprevalence of specific types of cutaneous HPV by gender [35]. Our findings are generalizable to non-Hispanic Whites only. However, our study population of Florida residents is at higher risk of NMSC compared to other U.S. states, corresponding to Florida's higher UV radiation index [42], and hence may represent a population that could be targeted for novel NMSC prevention measures. To maximize the sample size, persistence of HPV infection was determined using both incident and prevalent HPV infections. Therefore, the true duration of HPV infection is unknown, given the prevalent cases at baseline. Finally, we examined only 25 HPV types in genus β and 16 HPV types in genus γ. Given the large number of HPV types that are being continually discovered, our results on overall β and γ HPV infection may not reflect the true associations that can be affected by unexamined and unknown HPV types.

The study has several strengths. This is the first study to report HPV infection across a continuum of incidence, prevalence, persistence and clearance of cutaneous HPV across different genera, by type and species. We compared HPV infection in normal skin swabs as well as eyebrow hairs, thus providing a comprehensive report on natural history of HPV using biomarkers from two tissues. This is particularly important since concordance between HPV infection in normal skin swabs and eyebrow hairs was found to vary by HPV type. Along with rates of HPV infection, we also evaluated a range of epidemiological factors associated with HPV infection, overall and by species. Analyses were adjusted for potential confounders.

In conclusion, we observed high prevalence, incidence and persistence of cutaneous HPV infection among cancer-free men. Age and history of blistering sunburns were significantly associated with prevalence and persistence of β HPV. The association of blistering sunburns with both persistence of HPV infection, but not with incidence, suggests that sunlight exposure does not affect acquisition of new HPV infection but the duration of HPV infection could be affected by sunlight exposure through UV related immune dysregulation or actual promotion of HPV infection. Given the potential role of cutaneous β HPV in NMSC, the findings are valuable in defining a high risk population for development of novel preventive measures.

## References

[1] Braaten KP, Laufer MR (2008) Human Papillomavirus (HPV), HPV-Related Disease, and the HPV Vaccine. Rev Obstet Gynecol 1: 2–10. 18701931 PMC2492590

[2] Chiantore MV, Vannucchi S, Accardi R, Tommasino M, Percario ZA, et al (2012) Interferon-beta induces cellular senescence in cutaneous human papilloma virus-transformed human keratinocytes by affecting p53 transactivating activity. PLoS One 7: 16. 10.1371/journal.pone.0036909 PMC3353995 22615843

[3] Bzhalava D, Guan P, Franceschi S, Dillner J, Clifford G (2013) A systematic review of the prevalence of mucosal and cutaneous human papillomavirus types. Virology 445: 224–231. 23928291 10.1016/j.virol.2013.07.015

[4] Heideman DA, Waterboer T, Pawlita M, Delis-van Diemen P, Nindl I, et al (2007) Human papillomavirus-16 is the predominant type etiologically involved in penile squamous cell carcinoma. J Clin Oncol 25: 4550–4556. 17925550 10.1200/JCO.2007.12.3182

[5] Smith JS, Lindsay L, Hoots B, Keys J, Franceschi S, et al (2007) Human papillomavirus type distribution in invasive cervical cancer and high-grade cervical lesions: a meta-analysis update. Int J Cancer 121: 621–632. 17405118 10.1002/ijc.22527

[6] Castellsague X, Pawlita M, Roura E, Margall N, Waterboer T, et al (2013) Prospective seroepidemiologic study on the role of Human Papillomavirus and other infections in cervical carcinogenesis: Evidence from the EPIC cohort. Int J Cancer 13: 28665. 10.1002/ijc.28665 24338606

[7] Michaud DS, Langevin SM, Eliot M, Nelson HH, Pawlita M, et al (2014) High-risk HPV types and head and neck cancer. Int J Cancer 26: 28811. 10.1002/ijc.28811 PMC4107082 24615247

[8] Mansour M, Touka M, Hasan U, Bellopede A, Smet A, et al (2007) E7 properties of mucosal human papillomavirus types 26, 53 and 66 correlate with their intermediate risk for cervical cancer development. Virology 367: 1–9. 17568647 10.1016/j.virol.2007.05.005

[9] Pecoraro G, Morgan D, Defendi V (1989) Differential effects of human papillomavirus type 6, 16, and 18 DNAs on immortalization and transformation of human cervical epithelial cells. Proc Natl Acad Sci U S A 86: 563–567. 2463631 10.1073/pnas.86.2.563 PMC286512

[10] Proby CM, Harwood CA, Neale RE, Green AC, Euvrard S, et al (2011) A case-control study of betapapillomavirus infection and cutaneous squamous cell carcinoma in organ transplant recipients. Am J Transplant 11: 1498–1508. 21718442 10.1111/j.1600-6143.2011.03589.x

[11] Struijk L, Bouwes Bavinck JN, Wanningen P, van der Meijden E, Westendorp RG, et al (2003) Presence of human papillomavirus DNA in plucked eyebrow hairs is associated with a history of cutaneous squamous cell carcinoma. J Invest Dermatol 121: 1531–1535. 14675206 10.1046/j.1523-1747.2003.12632.x

[12] Neale RE, Weissenborn S, Abeni D, Bavinck JNB, Euvrard S, et al (2013) Human Papillomavirus Load in Eyebrow Hair Follicles and Risk of Cutaneous Squamous Cell Carcinoma. Cancer Epidemiology Biomarkers & Prevention 22: 719–727. 10.1158/1055-9965.EPI-12-0917-T 23396961

[13] Jackson S, Storey A (2000) E6 proteins from diverse cutaneous HPV types inhibit apoptosis in response to UV damage. Oncogene 19: 592–598. 10698529 10.1038/sj.onc.1203339

[14] Muschik D, Braspenning-Wesch I, Stockfleth E, Rosl F, Hofmann TG, et al (2011) Cutaneous HPV23 E6 prevents p53 phosphorylation through interaction with HIPK2. PLoS One 6: 16. 10.1371/journal.pone.0027655 PMC3218003 22110707

[15] Jablonska S, Dabrowski J, Jakubowicz K (1972) Epidermodysplasia Verruciformis as a Model in Studies on the Role of Papovaviruses in Oncogenesis. Cancer Research 32: 583–589. 5061309

[16] Rollison DE, Pawlita M, Giuliano AR, Iannacone MR, Sondak VK, et al (2008) Measures of cutaneous human papillomavirus infection in normal tissues as biomarkers of HPV in corresponding nonmelanoma skin cancers. Int J Cancer 123: 2337–2342. 18729188 10.1002/ijc.23795

[17] Farzan SF, Waterboer T, Gui J, Nelson HH, Li Z, et al (2013) Cutaneous alpha, beta and gamma human papillomaviruses in relation to squamous cell carcinoma of the skin: A population-based study. Int J Cancer 28: 28176. 10.1002/ijc.28176 PMC3713187 23536363

[18] Iannacone MR, Gheit T, Waterboer T, Giuliano AR, Messina JL, et al (2012) Case-control study of cutaneous human papillomaviruses in squamous cell carcinoma of the skin. Cancer Epidemiol Biomarkers Prev 21: 1303–1313. 22707711 10.1158/1055-9965.EPI-12-0032 PMC4543310

[19] Andersson K, Michael KM, Luostarinen T, Waterboer T, Gislefoss R, et al (2012) Prospective study of human papillomavirus seropositivity and risk of nonmelanoma skin cancer. Am J Epidemiol 175: 685–695. 22419740 10.1093/aje/kwr373

[20] Iannacone MR, Gheit T, Waterboer T, Giuliano AR, Messina JL, et al (2013) Case-control study of cutaneous human papillomavirus infection in Basal cell carcinoma of the skin. J Invest Dermatol 133: 1512–1520. 23303448 10.1038/jid.2012.478 PMC4545594

[21] de Koning MN, Struijk L, Bavinck JN, Kleter B, ter Schegget J, et al (2007) Betapapillomaviruses frequently persist in the skin of healthy individuals. J Gen Virol 88: 1489–1495. 17412978 10.1099/vir.0.82732-0

[22] Boxman IL, Berkhout RJ, Mulder LH, Wolkers MC, Bouwes Bavinck JN, et al (1997) Detection of human papillomavirus DNA in plucked hairs from renal transplant recipients and healthy volunteers. J Invest Dermatol 108: 712–715. 9129220 10.1111/1523-1747.ep12292090

[23] Antonsson A, Karanfilovska S, Lindqvist PG, Hansson BG (2003) General acquisition of human papillomavirus infections of skin occurs in early infancy. J Clin Microbiol 41: 2509–2514. 12791874 10.1128/JCM.41.6.2509-2514.2003 PMC156491

[24] Hsu JY, Chen AC, Keleher A, McMillan NA, Antonsson A (2009) Shared and persistent asymptomatic cutaneous human papillomavirus infections in healthy skin. J Med Virol 81: 1444–1449. 19551818 10.1002/jmv.21529

[25] Antonsson A, Erfurt C, Hazard K, Holmgren V, Simon M, et al (2003) Prevalence and type spectrum of human papillomaviruses in healthy skin samples collected in three continents. J Gen Virol 84: 1881–1886. 12810883 10.1099/vir.0.18836-0

[26] Anic GM, Lee J-H, Stockwell H, Rollison DE, Wu Y, et al (2011) Incidence and human papillomavirus (HPV) type distribution of genital warts in a multinational cohort of men: the HPV in men study. Journal of Infectious Diseases 204: 1886–1892. 22013227 10.1093/infdis/jir652 PMC3209812

[27] Giuliano AR, Lee JH, Fulp W, Villa LL, Lazcano E, et al (2011) Incidence and clearance of genital human papillomavirus infection in men (HIM): a cohort study. Lancet 377: 932–940. 21367446 10.1016/S0140-6736(10)62342-2 PMC3231998

[28] Gheit T, Billoud G, de Koning MN, Gemignani F, Forslund O, et al (2007) Development of a sensitive and specific multiplex PCR method combined with DNA microarray primer extension to detect Betapapillomavirus types. J Clin Microbiol 45: 2537–2544. 17581938 10.1128/JCM.00747-07 PMC1951219

[29] Gheit T, Landi S, Gemignani F, Snijders PJ, Vaccarella S, et al (2006) Development of a sensitive and specific assay combining multiplex PCR and DNA microarray primer extension to detect high-risk mucosal human papillomavirus types. J Clin Microbiol 44: 2025–2031. 16757593 10.1128/JCM.02305-05 PMC1489390

[30] Schmitt M, Dondog B, Waterboer T, Pawlita M, Tommasino M, et al (2010) Abundance of Multiple High-Risk Human Papillomavirus (HPV) Infections Found in Cervical Cells Analyzed by Use of an Ultrasensitive HPV Genotyping Assay. Journal of Clinical Microbiology 48: 143–149. 19864475 10.1128/JCM.00991-09 PMC2812266

[31] Iannacone MR, Gheit T, Pfister H, Giuliano AR, Messina JL, et al (2013) Case-control study of genus-beta human papillomaviruses in plucked eyebrow hairs and cutaneous squamous cell carcinoma. Int J Cancer 17: 28552. 10.1002/ijc.28552 PMC4545525 24136717

[32] Viera AJ, Garrett JM (2005) Understanding interobserver agreement: the kappa statistic. Fam Med 37: 360–363. 15883903

[33] Sampogna F, Bavinck JNB, Pawlita M, Abeni D, Harwood CA, et al (2012) Factors associated with the seroprevalence of 26 cutaneous and two genital human papillomavirus types in organ transplant patients. Journal of General Virology 93: 165–174. 21900419 10.1099/vir.0.035493-0

[34] Antonsson A, Green AC, Mallitt KA, O'Rourke PK, Pandeya N, et al (2010) Prevalence and stability of antibodies to 37 human papillomavirus types—a population-based longitudinal study. Virology 407: 26–32. 20723959 10.1016/j.virol.2010.07.046

[35] Iannacone MR, Michael KM, Giuliano AR, Waterboer T, Pawlita M, et al (2010) Risk factors for cutaneous human papillomavirus seroreactivity among patients undergoing skin cancer screening in Florida. Journal of Infectious Diseases 201: 760–769. 20105078 10.1086/650466

[36] de Koning MNC, Weissenborn SJ, Abeni D, Bouwes Bavinck JN, Euvrard S, et al (2009) Prevalence and associated factors of betapapillomavirus infections in individuals without cutaneous squamous cell carcinoma. Journal of General Virology 90: 1611–1621. 19321753 10.1099/vir.0.010017-0

[37] Schneider I, Lehmann MD, Kogosov V, Stockfleth E, Nindl I (2013) Eyebrow hairs from actinic keratosis patients harbor the highest number of cutaneous human papillomaviruses. BMC Infect Dis 13: 1471–2334. 10.1186/1471-2334-13-186 PMC3642014 23618013

[38] Escutia B, Ledesma E, Serra-Guillen C, Gimeno C, Vilata JJ, et al (2011) Detection of human papilloma virus in normal skin and in superficial and nodular basal cell carcinomas in immunocompetent subjects. Journal of the European Academy of Dermatology and Venereology 25: 832–838. 21054564 10.1111/j.1468-3083.2010.03875.x

[39] Termorshuizen F, Feltkamp MC, Struijk L, de Gruijl FR, Bavinck JN, et al (2004) Sunlight exposure and (sero)prevalence of epidermodysplasia verruciformis-associated human papillomavirus. J Invest Dermatol 122: 1456–1462. 15175037 10.1111/j.0022-202X.2004.22617.x

[40] Iannacone MR, Wang W, Stockwell HG, O'Rourke K, Giuliano AR, et al (2012) Sunlight Exposure and Cutaneous Human Papillomavirus Seroreactivity in Basal Cell and Squamous Cell Carcinomas of the Skin. Journal of Infectious Diseases 206: 399–406. 22661119 10.1093/infdis/jis374 PMC4542577

[41] Chen AC, McMillan NA, Antonsson A (2008) Human papillomavirus type spectrum in normal skin of individuals with or without a history of frequent sun exposure. J Gen Virol 89: 2891–2897. 18931088 10.1099/vir.0.2008/003665-0

[42] EPA UV Index: United States Environmental Protection Agency.

